# Investigating the crowding effect of FDI on domestic investments: Evidence from Bangladesh

**DOI:** 10.1016/j.heliyon.2024.e31092

**Published:** 2024-05-10

**Authors:** Ai-Jun Guo, Sayed Farrukh Ahmed, A.K.M. Mohsin, Arifur Rahman, Shamsul Nahar Abdullah, Choo Wou Onn, Mohammad Saiyedul Islam

**Affiliations:** a School of Economics, Lanzhou University, Gansu, Lanzhou, China; b Faculty of Business & Entrepreneurship, Daffodil International University, Daffodil Smart City, Ashulia, Dhaka, Bangladesh; c Faculty of Business & Communications, INTI International University, Nilai, Malaysia; d Faculty of Data Science and Information Technology, INTI International University, Nilai, Malaysia; e School of International Trade and Economics, Jiangxi University of Finance and Economics Jiangxi, China

**Keywords:** Crowding effect, Domestic investments, Foreign direct investment (FDI), Vector error correction model (VECM)

## Abstract

This study empirically investigates the crowding effect of Foreign Direct Investment (FDI) on domestic investments in Bangladesh, utilizing annual time series data from 1972 to 2022. Initially, unit root tests are conducted with and without considering structural breaks in the dataset. This study employs the Johansen test of cointegration to investigate the enduring association between the variables and utilizes the Vector Error Correction Model (VECM) to accommodate this relationship over the long term. Following the estimation of the VECM, formulas about the magnitude of the crowding effect (CE) are applied to examine the impact of FDI on domestic investment in Bangladesh. Results indicate that FDI positively influences domestic investments in both the short and long run.

## 1 Introduction

Foreign Direct Investment (FDI) stands as a pivotal driver for economic advancement, competitiveness, employment generation, and export growth in host countries [[1], [2], [3], [4]]. Economies with abundant capital resources seek opportunities in foreign markets to maximize returns by leveraging the resources, talents, and markets of host nations [5,6]. Conversely, countries experiencing capital shortages often attract FDI to close their savings-investment gap, facilitate the transfer of technology and knowledge, and stimulate economic growth [[7], [8], [9]].

The influence of FDI on the economic growth of a host nation may be observed indirectly through its impact on domestic investment. FDI has the potential to either stimulate or impede domestic investments, known respectively as the crowding-in or crowding-out effect [10,11]. The crowding-in effect occurs when FDI encourages investments by local firms, whereas the crowding-out occurs when FDI disrupts investments by domestic entities.

This issue of the crowding effect of FDI on domestic investments has garnered significant scholarly attention in recent times [6,[12], [13], [14], [15], [16], [17], [18], [19], [20], [21]]. Some studies have concluded that FDI inflows have crowded-in domestic investments, while others have suggested a crowding-out effect.

Bangladesh, since gaining independence in 1971, has attracted FDI across various sectors including agriculture, textiles, and energy. Despite fluctuations in FDI inflows, Bangladesh has remained an attractive destination for foreign investment. Nevertheless, the effect of Foreign Direct Investment (FDI) on the economic growth of Bangladesh continues to be a topic of discussion. Studies by Refs. [8,22,23] have produced varied conclusions regarding the relationship between FDI and economic growth in Bangladesh, underscoring the need for further investigation. For a resource-scarce country like Bangladesh, attracting foreign investments becomes imperative, especially since large-scale investments are essential across numerous sectors and local funds are insufficient to meet those demands. The government has encouraged FDI through various policy measures such as tax holidays and exemptions, with the expectation of supplementing domestic investments. However, the issue of the crowding effect of FDI on domestic investments remains inadequately explored for a country like Bangladesh, leaving policymakers with a limited understanding of whether FDI has encouraged domestic investments or displaced investments made by domestic firms. Addressing this knowledge gap can provide insights into various aspects of foreign direct investment in Bangladesh and guide the development of effective policies.

The primary objective of this study is to examine the crowding effect of FDI on domestic investments in Bangladesh, aiming to discern whether FDI encourages or displaces domestic investment. By addressing this gap, the study contributes to the existing literature by providing insights into the relationship between foreign direct investment (FDI) and domestic investments in Bangladesh. It is anticipated that the findings will inform policymakers about the impact of FDI on domestic investments, thereby aiding in the formulation of strategies to attract FDI, with significant implications for domestic investment growth.

The paper is structured as follows: Section 2 provides an overview of the FDI scenarios in Bangladesh, followed by a review of related empirical literature in Section 3. Section 4 outlines the data and methodology employed in the study. Section 5 presents the results and subsequent discussion. Finally, Section 6 concludes the study.

## 2 Overview of FDI scenarios of Bangladesh

**Fig. 1:**
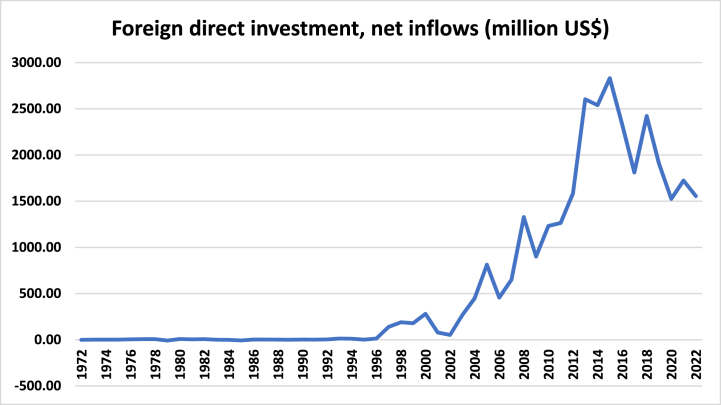
FDI net inflows (million US$) in Bangladesh (1972–2022).

Source: (WDI, 2023)

Fig. 1 shows that FDI net inflows in Bangladesh had been increasing from 1996. After sudden fall in 2002, the country witnessed the increasing trend of FDI net inflows. FDI net inflows had increased significantly in Bangladesh during 2009–2015.

After 2015, FDI net inflows decreased (except in 2018), a major concern for policymakers in Bangladesh. To attract more FDI, the government of Bangladesh has offered numerous fiscal and non-fiscal incentives to foreign investors, including 100 % foreign equity (except nuclear energy, defense, currency, and forest plantations), exemption of customs duties on machinery, and tax exemption on payment of interest on foreign loans [24].

## 3 Literature review

FDI may affect the economic growth of host countries indirectly by influencing domestic investment. Due to the entry of FDI in the host country, there may be the possibility of crowding-in (out) effect of FDI on domestic investment. The crowding-in effect of FDI occurs when the presence of FDI encourages domestic investments of host countries. In contrast, the crowding-out effect of FDI occurs when FDI distorts investments made by domestic firms and drives them out of the competition with foreign firms [10,15,25,26]. Additionally [27,28], suggests that crowding-in may occur due to technology and knowledge spillovers from foreign companies to domestic ones. Moreover, the entry of FDI into the host country can increase employment opportunities, thereby boosting local expenditure on locally produced goods, which could benefit domestic investors.

In contrast, crowding-out of domestic investment may happen in numerous ways [13]. Local companies might not be as competitive as foreign companies because foreign companies might be more experienced or efficient in producing products and selling them at comparatively cheaper prices than domestic firms. Besides, foreign investors can start hiring skilled or efficient workers away from domestic firms which may adversely affect the productivity of domestic firms. In literature, the issues of the crowding effect of FDI on domestic investments have been dealt with by many researchers [[4], [5], [6], [7],^10^,^11^,[13], [14], [15], [16], [17], [18], [19], [20],^25^,^29^].

Some studies [17] [4,6,10,18] [12,20,29] concluded that inflows of FDI into host countries have crowded-in domestic investments. On the other hand, some other studies [11,[13], [14], [15], [16],^19^] asserted that FDI has crowded-out domestic investments.

Numerous studies have investigated the correlation between FDI and domestic investment in diverse regions and elucidated the crowding effects of FDI on domestic investment. A study [30] focused on 23 Eastern European nations from 1991 to 2019, revealing a crowding-out effect of FDI on domestic investment in the region. They emphasized the necessity of initiatives to attract and maintain FDI in Eastern Europe.

A study conducted by Ref. [31] investigated the impact of foreign investment on domestic investment in Vietnam from 2010 to 2015, employing GMM estimation. Their findings indicated a positive correlation between FDI and domestic private investment, particularly in downstream sectors, suggesting a crowding-in effect. Similarly [32], explored South Asian economies from 1991 to 2021, using the FMOLS approach, concluding a crowding-in effect of FDI on domestic investment, highlighting the potential roles of financial inclusion, globalization, and macroeconomic stability.

Examining FDI's impact on domestic investment [33], analyzed the effect in BRICS countries from 1988 to 2020, employing the FMOLS approach. Their findings demonstrated a considerable increase in domestic investment due to FDI inflows. Similarly [34], focused on Jordan, employing the ARDL model from 1980 to 2017, revealing a crowding-out effect of FDI on domestic investment, particularly when multinational corporations enter industries formerly controlled by state-owned companies.

Exploring the relationship between FDI and domestic investment in Indonesia [35], examined Indonesia's FDI and private domestic investment relationship from 1990 to 2020. Their observations revealed a sectoral level crowding-in effect in primary and secondary sectors, while the tertiary sector showed a neutral association. In a related study [36], investigated FDI's impact on Indonesian domestic investment from 2010 to 2018, using the VECM, concluding a crowding-in relationship, and recommending governmental support to strengthen the investment environment.

Investigating the effects of FDI on domestic investment [37], explored the crowding impacts of FDI on Indian domestic investment from 1990 to 91 to 2014–15, revealing a crowding-out effect. Similarly [38], analyzed MENA countries' FDI and domestic investment relationship from 1981 to 2017, noting a long-term positive impact of FDI on domestic investment.

Examining the relationship between FDI and domestic investment across diverse regions [39], focused on fifteen SADC nations from 1991 to 2019. Employing the panel ARDL technique, their study revealed varied effects of FDI on domestic investment based on income levels, with higher-income SADC nations experiencing crowding out and lower-income nations showing no influence of FDI on domestic investment. These studies collectively provide insights into the complex dynamics of FDI and its implications for domestic investment across diverse regions.

Analyzing the relationship between sectoral FDI and domestic investment in Pakistan [12], using data from 1980 to 2012, applied the ARDL model. Their study concluded that aggregate FDI has a crowded-in effect on the domestic investment of Pakistan. At the sectoral level, the study found a crowding-in effect of manufacturing FDI and services FDI on the domestic investment of Pakistan.

Investigating the impact of FDI on domestic investment in various regions [16], delved into the crowding effect of FDI on domestic investment by utilizing data from selected Eastern European countries over the period from 1996 to 2018, employing panel data analysis. The findings of the study suggested the crowding-out effect of FDI on domestic investment in the short run, but the crowding-in effect on domestic investment in the long run. Similarly, a separate study [15], using data from 1982 to 2016 and applying the ARDL model, examined the relationship between FDI and domestic investment in China. The study indicated that FDI has a crowding-out effect on the domestic investment of China. Additionally, the study [10] examined the relationship between FDI and domestic investment considering data from 10 Central and Eastern European countries from the period 1995 to 2015, employing the GMM estimation method. The study discovered the crowding-out effect of FDI on domestic investment in the short run, while indicating a crowding-in effect of FDI on domestic investment in the long run.

Using data of 14 developing countries (including Bangladesh) between 1990 and 2017 and applying the panel DOLS estimation method [25], disclosed that FDI has crowding-out effect on the domestic investments of the developing countries chosen for the study. The study conducted by Ref. [14] used data of 30 Chinese provinces between 2000 and 2014 for examining the crowding effect of FDI on domestic investment. The findings of the study revealed the crowding-out effect of FDI on Chinese domestic investment. The study of [7], using data of 30 OECD countries during the period 2006–2013, investigated the impact of FDI on domestic investments through employing the GMM estimation method. The results of the study found no significant impact of FDI on domestic investments [4]. used the data for the period 1994Q1 to 2014Q4 in investigating the crowding effect of FDI on Chinese domestic investment by applying the ARDL model. Empirical findings of the study disclosed the crowding-in effect of equity joint venture on Chinese domestic investment, whereas in case of wholly foreign-funded enterprise, the study found crowding-out effect [6]. applied the GMM estimation system for exploring the impact of FDI on domestic investment by considering panel data of 46 countries over the periods of 1996 and 2009 and the study concluded that inflows of FDI had crowded-in domestic investment of the sample countries. In their study [19], using panel data from 46 developing countries between 1996 and 2009 and applying the system GMM estimation technique, argued that FDI is likely to crowd-out domestic investment mostly of those countries having better governance.

A study [29] used data from five South Asian countries from 1980 to 2010 to examine the crowding effect of FDI on domestic investment. The study confirmed the crowding-in effect of FDI on the domestic investment for India, Pakistan, Sri Lanka, and Bangladesh both in the long-run and short-run. Additionally the study [1], considering panel data of 26 European Union (EU) countries covering the period from 1990 to 2008 revealed that FDI crowded-out domestic investment for 14 EU economies (old member), whereas the study found no evidence that FDI crowded-out domestic investment for rest 12 EU economies (new member).

Examining the relationship between FDI and domestic investment across different regions [21], applying the panel Fully Modified OLS (FMOLS) estimation method and utilizing panel data of nine Latin American countries over the years 1980–2002, showed that FDI had a crowded-in effect on domestic investment of the selected Latin American economies. In contrast [5], utilizing data of 50 countries between the periods 1970 and 2004 and using various panel estimation methods, disclosed that FDI is likely to crowd-out domestic investment in developed countries, whereas the impact of FDI is found to be neutral in Less Developed Countries (LDCs).

Examining the relationship between FDI and domestic investment in various regions, [20]; utilized a panel fixed-effect model to explore the relationship between FDI and domestic investment by using panel data from 38 Sub-Saharan African (SSA) nations from 1970 to 2005. The study found that FDI had a crowded-in effect on domestic investment for the selected countries. Additionally [17], using panel data of 29 mainland provinces of China from 1987 to 2001, indicated that the inflows of FDI have crowded-in impacts on domestic investments of China. The author also stated that the Chinese government's policies of attracting FDI to form joint ventures with local enterprises led to the improvement of their technological level and advancements in the production process.

Exploring the dynamics between FDI and domestic investments [11], utilized annual data from 30 countries over the period from 1992 to 2002 to investigate the relationship by applying panel cointegration techniques. The authors opined that host firms with comparatively low-quality products cannot compete properly with foreign firms and, in turn, prolific foreign firms may crowd out the least efficient domestic competitors. Moreover [13], considering panel data of 36 countries from Asia, Africa, and Latin America covering 1971–2000 found that FDI crowded-out domestic investment for the Latin American countries.

Examining the crowding effects of FDI on investments made by domestic companies [18], investigated using data from selected countries of Asia, Africa, and Latin America for 1970 and 1996. The study found that FDI had crowding-in effects in the selected Asian and African countries but crowding-out effects in the selected Latin American countries.

The literature reviewed, as outlined in Table 1, demonstrates that empirical evidence regarding the crowding effects of Foreign Direct Investment (FDI) on domestic investment in host countries is inconclusive. While certain studies [4,6,10,12,17,18,20,21] suggest that FDI may promote domestic investment, thereby fostering economic growth through a crowding-in effect, others [1,5,11,[13], [14], [15], [16],^19^] argue that FDI could impede growth via a crowding-out effect. Importantly, the specific dynamics of the crowding effect of FDI on domestic investments remain largely unexplored in the context of Bangladesh.

**Table 1:** Crowding effects of FDI on Domestic investment: Selected Empirical Evidence.

Country	Author(s)	Study Period	Major Findings
Eastern European countries	[30]	1991–2019	FDI has a crowding-out effect on domestic investment in Eastern European countries in the short-run while exhibiting a crowding-in effect in the long-run. Initiatives to attract and retain FDI are imperative.
Vietnam	[31]	2010–2015	FDI in Vietnam positively motivates domestic private investment, especially in downstream sectors, indicating a crowding-in effect.
BRICS countries	[33]	1988–2020	FDI inflow significantly increases domestic investment in BRICS countries, necessitating strategies to enhance FDI to boost domestic investment.
Indonesia	[35]	1990–2020	In Indonesia, the primary and secondary sectors witness a crowding-in impact of FDI on domestic investment, whereas the tertiary sector demonstrates neutrality.
MENA countries	[38]	1981–2017	FDI in MENA countries has a long-term positive impact on domestic investment, especially between 2001 and 2017.
India	[37]	1990–2015	FDI in India exhibits a crowding-out effect on domestic investment.
Pakistan	[12]	1980–2012	Aggregate FDI has crowded-in effect on the domestic investment of Pakistan. At sectoral level, the study found crowding-in effect of manufacturing FDI and services FDI on the domestic investment
Selected Eastern European countries	[16]	1996–2018	Crowding-out effect of FDI on domestic investment in the short-run, while crowding-in effect on domestic investment in the long-run
China	[15]	1982–2016	Crowding-in effect of equity joint venture on Chinese domestic investment, whereas in the case of a wholly foreign-funded enterprise, the study found crowding-out effect
10 Central and Eastern European countries	[10]	1995–2015	Crowding-out effect of FDI on domestic investment in the short-run, while crowding-in effect of FDI on domestic investment in the long-run
14 Developing countries	[25]	1990–2017	FDI has crowding-out effect on the domestic investments of the developing countries chosen for the study
30 Chinese provinces	[14]	2000–2014	Crowding-out effect of FDI on Chinese domestic investment
30 OECD countries	[7]	2006–2013	No significant impact of FDI on domestic investments
China	[4]	1994Q1 -2014Q4	Crowding-in effect of equity joint venture on Chinese domestic investment, whereas in case of wholly foreign-funded enterprise, the study found crowding-out effect
46 countries	[6]	1996–2009	Inflows of FDI had crowded-in domestic investment of the sample countries
Five South Asian countries	[29]	1980–2010	Crowding-in effect of FDI on the domestic investment for India, Pakistan, Sri Lanka, and Bangladesh both in the long-run and short-run
46 developing countries	[19]	1996–2009	FDI is likely to crowd-out domestic investment mostly of those countries having better governance
26 European Union (EU) countries	[1]	1990–2008	FDI crowded-out domestic investment for 14 EU economies (old member)
9 Latin American countries	[21]	1980–2002	FDI had crowded-in domestic investment of the selected Latin American economies
50 countries	[5]	1970–2004	FDI is likely to crowd-out domestic investment in developed countries
38 Sub-Saharan African (SSA) nations	[20]	1970–2005	FDI had crowded-in domestic investment for the selected countries
29 mainland provinces of China	[17]	1987–2001	Inflows of FDI have crowded-in impacts on domestic investments of China
30 countries	[11]	1992–2002	Foreign firms may crowd-out least efficient domestic competitors
36 countries from Asia, Africa and Latin America	[13]	1971–2000	FDI crowded-out domestic investment for the Latin American countries.
Selected countries of Asia, Africa and Latin America	[18]	1970–1996	FDI had crowding-in effects in the selected Asian and African countries, but crowding-out effects in the selected Latin American countries

Although there is a wealth of existing literature, a systematic and rigorous review reveals a notable gap in the understanding of the specific dynamics of the crowding effect of FDI on domestic investment, particularly in the context of Bangladesh. While studies have explored this relationship in various countries and regions, there is a lack of comprehensive research focusing on Bangladesh.

This literature gap presents an opportunity for further research to provide clarity on whether FDI in Bangladesh encourages or hampers domestic investments. It is essential to address this gap to provide policymakers with accurate information about the impact of foreign direct investment (FDI) on domestic investment in Bangladesh. By understanding the underlying dynamics, policymakers can devise strategies to attract FDI effectively, potentially fostering domestic investment growth and overall economic development.

## 4 Data and methodology

### 4.1 Data

The study utilizes annual time series data from Bangladesh spanning the period from 1972 to 2022. The data for this timeframe were sourced from the World Development Indicators [40]. Detailed descriptions of the variables used in the analysis are provided in Table 2 below.

**Table 2:** Description of the variables.

Variables	Description
RGDP	RGDP represents the real gross domestic product (constant 2015 US$), serving as a metric to gauge the economic performance of Bangladesh [[41], [42], [43], [44]]
RFDI	RFDI represents the real foreign direct investment net inflows ratio to gross domestic product. This variable, as per [17,[43], [44], [45], [46], [47]] is employed to examine the correlation between FDI inflows and GDP. FDI net inflow data (current US$) has been adjusted to real values by dividing the current figures by the GDP deflator (2015 = 100), with 2015 serving as the base year, following the methodology outlined in Ref. [44].
RGCF	RGCF represents the Ratio of Real Gross Capital Formation to Real Gross Domestic Product. This study employs Gross Capital Formation as a proxy for Bangladesh's total investment, as indicated by BBS (2020, n.d.). Investment or gross capital formation encompasses both gross fixed capital formation and changes in inventories. This variable is adopted in accordance with previous studies by Refs. [[48], [49], [50]].

Note.

All the variables have been measured in real terms (constant 2015 US$) following [9,42,51].

All variables in this study were logarithmically transformed to mitigate scaling issues. This approach aids in smoothing out data fluctuations and reducing the impact of extreme values or outliers on the coefficients [52]. Due to the occurrence of negative observations in RFDI, the variable transforms the Inverse Hyperbolic Sine (IHS) equation, as described by Equation (1) [13,33,53,54]:

(1) $$y=\ln\left(x+\sqrt{\left(x^{2}+1\right)}\right)$$

where, is the variable to be transformed and y is the new transformed variable.

### 4.2 Methodology

This study employs standard time series techniques to examine the crowding effect of FDI on domestic investments in Bangladesh. The testing procedure involves three steps: (i) assessing the stationarity of each time series variable, (ii) determining the presence of a long-run cointegrating relationship between the studied variables, and (iii) estimating a VECM if a long-run cointegrating relationship exists.

To address the issue of structural breaks, the study utilizes appropriate unit root tests such as ZA and LP. Conventional unit root tests like ADF and PP have limited ability to test the stationarity properties of each variable in the presence of structural breaks.

### 4.3 Unit root test without structural breaks

#### 4.3.1 ADF unit root test

The unit root test developed by the ADF can be employed to assess whether each variable possesses a unit root. The analysis involves comparing the null hypothesis of non-stationarity with the alternative hypothesis of stationarity [55].

The ADF unit root test is based on Equation (2):

(2) $$\Delta y_{t}=\mu +\beta t+\alpha y_{t-1}+\sum_{i=1}^{k}c_{i}\Delta y_{t-i}+\varepsilon_{t}$$

where, $y_{t}$ = the time series being tested; Δ = first difference operator; t = a time trend variable.

$\varepsilon_{t}$ = white noise error term.

#### 4.3.2 PP unit root test

PP unit root test can be applied when its stated statistics are strong for serial correlation and heteroscedasticity in which the null of non-stationarity has been tested against the alternative of stationarity [56].

The PP unit root test is based on Equation (3):

(3) $$\Delta y_{t}=\alpha +\theta y_{t-1}+\varepsilon_{t}$$

where, $\varepsilon_{t}$ (white noise error term) is *I* (0) and heteroskedastic. The test corrects the t-statistic of the coefficient (θ) from the model.

### 4.4 Unit root Test with structural breaks

The structural breaks issue in time series data have been a subject of widespread investigation that may occur for variety of reasons including financial or economic crises, regime shifts, and policy changes. Perron (1989) [57] argued that the results of the ADF and PP unit root tests could be biased towards rejecting the non-stationarity of the data if there are structural changes present in the data. To determine whether the data is stationary in the presence of structural breaks, unit root tests such as ZA and LP unit root tests have been employed.

#### 4.4.1 ZA unit root Test with one structural break

This analysis considers only a structural break determined from within the system, where the unit root hypothesis is tested against the alternative of no unit root [58]. The test includes three models, represented by Equations (4), (5), (6):

(4) $$\;\text{Model}\;A:\Delta y_{t}=\mu +\alpha_{1}y_{t-1}+\beta t+\theta_{1}DU_{t}+\sum_{j=1}^{k}d_{j}\Delta y_{t-j}+\varepsilon_{t}$$

(5) $$\;\text{Model}\;B:\Delta y_{t}=\mu +\alpha_{1}y_{t-1}+\beta t+\gamma_{1}DT_{t}+\sum_{j=1}^{k}d_{j}\Delta y_{t-j}+\varepsilon_{t}$$

(6) $$\;\text{Model}\;C:\Delta y_{t}=\mu +\alpha_{1}y_{t-1}+\beta t+\theta_{1}DU_{t}+\gamma_{1}DT_{t}+\sum_{j=1}^{k}d_{j}\Delta y_{t-j}+\varepsilon_{t}$$

Models A, B, and C represent three different scenarios: Model A shows a single change in the intercept, Model B shows a single change in the trend and Model C shows changes in both the intercept and trend. Here, two dummy variables represent a change in the intercept and a change in the trend occurring at the time break (TB).

#### 4.4.2 LP unit root test with two structural breaks

If there is more than one structural break in the data, consideration of a break can be insufficient which may lead to loss of information. Thus, the LP unit root test has been applied to capture two unknown structural breaks [59].

The LP unit root test is based on Equation (7):

(7) $$\Delta y_{t}=\mu +\alpha_{1}y_{t-1}+\beta t+\theta DU_{1t}+\gamma DT_{1t}+\omega DU_{2t}+\varphi DT_{2t}+\sum_{i=1}^{k}c_{i}\Delta y_{t-i}+\varepsilon_{t}$$

In equation (7), two dummy variables, $DU_{1t}$ and $DU_{2t}$, account for structural changes in the intercept at time break 1 (*TB1)* and time break 2 (*TB2)*, respectively; whereas two dummy variables, $DT_{1t}$ and $DT_{2t}$, account for shifts in the trend variable at time *TB1* and *TB2,* respectively.

If it has been determined that the selected variables are integrated in the same order, *I(1)*, confirmed by ZA and LP unit root tests, the long-run cointegrating relationship between the variables can be investigated by applying the Johansen cointegration test. Cointegration suggests the presence of a linear combination of stationary and nonstationary variables [60]. Before applying the Johansen cointegration test, the appropriate lag length has to be selected based on the LR test statistic, AIC, SC, FPE, and HQ Information Criterion.

The study [61] introduced two likelihood ratio tests, as delineated by Equations (8), (9) below:

(8) $$\lambda_{trace}\left(r\right)=-T\;\sum_{i=r+1}^{n}\ln\left(1-{\hat{\lambda }}_{r+1}\right)$$

(9) $$\lambda_{\max}\left(r,r+1\right)=-T\;\ln\left(1-{\hat{\lambda }}_{r+1}\right)$$

where, ${\hat{\lambda }}_{r+1}$ denotes the estimated eigenvalue of the characteristic roots; *r* = 0,1, 2, …and *T* = number of observations.

If the Johansen cointegration test verifies the existence of a long-term cointegrating relationship among the chosen variables, it is possible to estimate the Vector Error Correction Model (VECM) [62]. The VECM can assess the rate at which short-term dynamics adjust towards the long-term equilibrium when the variables are cointegrated [63].

Following [62,64,65], the VECM can be expressed as:

(10) $$\Delta LRGCF_{t}=\alpha_{3}+\sum_{i=1}^{k}\beta_{3i}\Delta LRGDP_{t-i}+\sum_{i=1}^{k}\gamma_{3i}\Delta LRFDI_{t-i}+\sum_{i=1}^{k}\theta_{3i}\Delta LRGCF_{t-i}+\rho_{3}ECT_{t-1}+\varepsilon_{3t}$$

Equation (10), Δ denotes the first difference operator. ΔLRGDP, ΔLRFDI and ΔLRGCF denote the differences in these variables capturing short-run disturbances. ECT (error correction term) captures the long-run effects [62]. $\rho_{3}$ is error correction coefficient. In addition, $\varepsilon_{3t}$ is a white-noise disturbance term.

This study is an attempt to examine the issues of the crowding effect of FDI on domestic investments in Bangladesh to identify whether FDI in Bangladesh encourages domestic investments or displaces the investments made by domestic firms. If FDI crowds in domestic investments, total investment is increased by more than the increase in FDI. On the other hand, if FDI crowds out domestic investments, total investment is increased by less than the increase in FDI. If FDI does not affect domestic investments, the total investment will be increased accordingly by the amount of FDI [13,17,18].

Following [13,17,18,66], the following formulas have been used in investigating the issues of the crowding effect of FDI on domestic investment:

In the long run, the magnitude of crowding effect (CE): δ−1. After running the model (see equation (10)), the coefficient of LRFDI in the long run can be generated. If the coefficient can be represented as δ and if, δ>1, RFDI has a positive effect on domestic investments (crowding-in effect) If, δ<1, RFDI has a negative effect on domestic investments (crowding-out effect)

If, δ=1, RFDI does not affect domestic investments (neutral effect). Where, δ is the coefficient of LRFDI in the long run.

In the short run, the magnitude of crowding effect (CE): $\sum \gamma_{3i}/\left(1-\sum \theta_{3i}\right)$.

Where, $\gamma_{3i}$ is the coefficient of LRFDI in short-run and $\theta_{3i}$ is the coefficient of LRGCF in short-run (see equation (10)).

If the magnitude of the crowding effect (CE) = 1, there is no effect of RFDI on domestic investments.

If the magnitude of the crowding effect (CE) > 1, RFDI crowds in domestic investments.

If the magnitude of the crowding effect (CE) < 1, RFDI crowds out domestic investments.

## 5 Results and discussion

Table 3 displays the quantitative characteristics of the variables. The average RGDP throughout the study period is US$101.76 billion, ranging from US$26.75 billion to US$305.52 billion. In contrast, the average RFDI during the study period was 0.39 percent, with a range spanning from −0.05 percent to 1.74 percent.

**Table 3:** Descriptive statistics of the variables.

Var.	Mean	Max.	Min.	Std. Dev.
RGDP	101.76	305.52	26.75	50.68
RFDI	0.39	1.74	−0.05	0.52
RGCF	18.75	32.73	1.48	8.06

The average RGCF is 18.75 percent. It ranges from 1.48 percent to 32.73 percent. The standard deviations indicate higher dispersion in data for RGDP compared to RFDI and RGCF.

Table 4 presents the results of the unit root test without structural break. From the results of the ADF and PP unit root test, it is apparent that LRGDP and LRFDI are non-stationary at level but stationary at the first difference, i.e. *I*(1). But, LRGCF is stationary at level, i.e. *I*(0).

**Table 4:** Results of unit root test without structural break.

Variables	ADF Test				PP Test				Order of Integration
	Intercept		Intercept and Trend		Intercept		Intercept and Trend		
	Level	1st diff.	Level	1st diff.	Level	1st diff.	Level	1st diff.	
LRGDP	3.98	0.63	0.35	−11.01^a^	9.50	−7.52^a^	2.47	−11.96^a^	I(1)
LRFDI	−1.64	−3.08^b^	−2.73	−2.97	−1.46	−8.49^a^	−2.71	−8.32^a^	I(1)
LRGCF	−6.61^a^	−6.13^a^	−8.9^a^	−6.34^a^	−6.51^a^	−6.13^a^	−9.91^a^	−7.21^a^	I(0)

*Notes*.

a

b denote significance at 1 % and 5 % levels respectively.

Table 5 shows the results of unit root tests with structural breaks. The results of the ZA unit root test show that LRGDP, LRFDI, and LRGCF are non-stationary at level but stationary at the first difference, i.e. *I*(1), in the presence of one structural break in the data.

**Table 5:** Results of unit root tests with structural breaks.

Variables	ZA Test		LP Test	
	t-statistic	TB	t-statistic	TB
LRGDP	−2.09	1998:01	−5.14	1988:01; 2002:01
Δ LRGDP	−11.71^a^	1989:01	−11.93^a^	1989:01; 2004:01
LRFDI	−4.24	2003:01	−5.15	1993:01; 2011:01
Δ LRFDI	−9.45^a^	2011:01	−10.27^a^	2002:01; 2011:01
LRGCF	−5.34	2011:01	−5.15	1981:01; 1995:01
Δ LRGCF	−6.26^a^	1992:01	−10.14^a^	1982:01; 1992:01

Note.

a denotes significance at 1 percent level.

The results of the LP unit root test also show that LRGDP, LRFDI, and LRGCF are non-stationary at level but stationary at the first difference, i.e. *I*(1), in the presence of two structural breaks in the data. As all the variables are integrated of order one, *I*(1), with breaks, (confirmed by ZA and LP unit root tests), the [61] test of cointegration has been applied to determine whether three variables are co-integrated.

Before the application of the Johansen cointegration test, the optimal lag has to be specified. Based on the LR test statistic, FPE, and AIC, an optimal lag of five has been found for the cointegration test (Table 6).

**Table 6:** Lag length selection.

Lag	LR	FPE	AIC	SC	HQ
0	NA	3.63e-08	−8.618472	−8.595594	−8.573159
1	443.4986	6.36e-13	−19.57163	−19.08015*	−19.39034*
2	14.36501	6.53e-13	−19.55205	−18.69193	−19.23486
3	0.845649	9.84e-13	−19.15907	−17.93033	−18.70595
4	10.21399	1.10e-12	−19.08093	−17.48356	−18.49187
5	36.48653*	4.57e-13*	−20.01318*	−18.04759	−19.28858

*Note.*

indicates lag order selected by the criterion.

In Table 7, trace statistics and maximum eigenvalue statistics disclose that three variables, e.g., LRGDP, LRFDI, and LRGCF have long-run equilibrium relationship or cointegrating relation indicating that in the long-term, LRGDP, LRFDI, and LRGCF move together.

**Table 7:** Results of johansen cointegration test.

Hypothesized No. of Co-integrating Equation (CE)	H_0_:	H_1_:	Eigen Value	Trace Test			Maximum Eigen Value Test		
				$\lambda_{trace}$	5 % Critical value	Prob.	$\lambda_{\max}$	5 % Critical value	Prob.
None^a^	r = 0	r = 1	0.5225	47.621	29.794	0.000	31.047	21.131	0.001
At most 1^a^	r ≤ 1	r = 2	0.3017	16.567	15.493	0.034	15.086	14.264	0.037
At most 2	r ≤ 2	r = 3	0.0345	1.477	3.841	0.224	1.477	3.841	0.224

*Note:* ‘*r*’ denotes the number of cointegrating vectors. Trace test indicates 2 cointegrating equation(s) at the 0.05.

level. Max-eigenvalue test indicates 2 cointegrating equation(s) at the 0.05 level.

a denotes rejection of the hypothesis at 5 % level.

Although both trace statistics and maximum eigen value statistics suggest to use two cointegrating equations, the present study uses only one cointegrating equation for avoiding complexity and ensuring simplicity for better understanding as suggested by Ref. [67]. As three variables have been co-integrated in the long-run, the VECM has been estimated which is used for identifying the crowding effect of FDI on domestic investment of Bangladesh.

Table 8 shows the long-run and short-run results of the magnitude of crowding effect of RFDI in Bangladesh. Based on the estimated output (please see appendix 1, appendix 2 and also follow the note 2), it is apparent that the magnitude of crowding effect of RFDI is found to be 25.73 in long-run which indicates that RFDI has crowding-in effect on the investments made by the domestic firms of Bangladesh in long-run. Additionally, the magnitude of crowding effect of RFDI is found to be 61.15 in short-run, which indicates that RFDI has crowding-in effect on the investments made by the domestic firms of Bangladesh in short-run. The results, thus, imply that total investment will be increased by more than the RFDI or one additional dollar of RFDI leads to more than a one-dollar increase in total investment.

**Table 8:** The long-run and short-run results of the magnitude of Crowding effect.^2^.

	Long-run	Short-run
Magnitude of Crowding effect	25.73	61.15
Remark	Crowding-in effect	Crowding-in effect

The findings of this study reveal a compelling insight into the relationship between Foreign Direct Investment (FDI) and domestic investment in Bangladesh. Contrary to some previous studies, the results suggest a crowding-in effect of FDI on investments made by domestic firms in Bangladesh, evident both in the short-run and long-run. This outcome underscores the potential of FDI to indirectly contribute to Bangladesh's economic growth by stimulating domestic investment.

One probable explanation for this crowding-in effect could be attributed to the technological spillover and operational enhancements facilitated by advanced foreign investments. As foreign firms bring in advanced technology and operational practices, domestic firms may benefit from technological transfers and improvements in their operations, thereby enhancing their capacity for further investments. This finding resonates with similar studies conducted in various contexts [4,6,10,12,17,18,20,21], highlighting the generalizability of the crowding-in effect of FDI on domestic investment.

The implications of these findings extend to policymakers, who play a crucial role in formulating and implementing policies to attract and leverage FDI effectively. Policymakers must not only devise pragmatic policies but also ensure their efficient implementation to maximize the benefits of FDI in Bangladesh's economy. Strengthening the capacity of governmental agencies to provide support services to foreign investors and ensuring seamless coordination between various government departments are imperative steps in this regard.

Moreover, policymakers can explore strategies to enhance the collaboration between foreign and domestic firms, particularly in sectors where domestic investors lack experience and technological expertise. By facilitating joint ventures and technology transfers from foreign to domestic firms, policymakers can bolster the efficiency and competitiveness of domestic enterprises, ultimately driving domestic investment.

From a policy perspective, the government may consider incentivizing foreign investors to collaborate with local firms through privileged treatment policies, such as joint ventures, aimed at promoting technological spillovers and knowledge transfers [68]. Financial and non-financial support mechanisms should be extended to domestic companies willing to engage in joint ventures with foreign investors, fostering a conducive environment for collaboration [69,70].

Furthermore, policymakers can devise strategies that capitalize on the backward and forward linkages of FDI. Encouraging FDI in sectors that rely on local suppliers for inputs can stimulate domestic investments by creating opportunities for local firms to serve the demand of foreign firms. Additionally, promoting FDI in the production of high-quality products can enhance the competitiveness of local firms by providing them with access to locally produced inputs, thus fostering their profitability and success.

## 6 Conclusion & policy implications

This study examines the crowding effect of foreign direct investment (FDI) on domestic investments in Bangladesh. It utilizes annual time series data spanning from 1972 to 2022. The Unit root tests were performed with and without accounting for potential structural breaks in the dataset. The Johansen cointegrations have been utilized to ascertain the presence of cointegration among the variables. The results of the Johansen cointegration indicate that the variables LRGDP, LRFDI, and LRGCF exhibit a long-run equilibrium relationship or cointegrating relation. It suggests that LRGDP, LRFDI, and LRGCF exhibit a positive correlation over a prolonged period and tend to change in the same direction. The estimation of the VECM has been conducted due to the long-run co-cointegration of three variables. Following the estimation of the VECM, researchers have utilized formulas to examine the magnitude of the crowding effect (CE) and its impact on domestic investment in Bangladesh.

The study's theoretical findings suggest that RFDI has a crowding-in effect on domestic investments in Bangladesh, which is evident in both the long and short terms. It implies that RFDI may indirectly influence Bangladesh's economic growth by bolstering domestic investment. According to the extant literature, one possible explanation might be the foreign firms with advanced technology may contribute to the improvement of the local firms' technological base through technology transfer, thus, local firms gain access to latest and advanced technology, increase their productivity, go for more production which may lead to economic growth.

The empirical results hold substantial importance for policymakers in Bangladesh, underscoring the necessity for strategic measures to leverage the favorable influence of FDI on domestic investment. This implies that policies geared towards integrating local firms into FDI-backed endeavors could strengthen the collaborative dynamic between foreign and domestic businesses. Furthermore, efforts to enhance the capacities and competitiveness of local enterprises are advisable, as they can augment the beneficial effects of FDI on domestic investment, thereby stimulating overall economic growth.

Furthermore, the practical implications of this study underscore the importance of policies that promote collaboration between foreign and domestic firms, particularly in sectors where technological expertise is lacking among domestic investors. Policymakers should prioritize initiatives aimed at facilitating technology transfer and knowledge sharing between foreign and domestic enterprises to maximize the developmental impact of FDI on Bangladesh's economy.

Overall, this study contributes to the existing literature by highlighting the crowding-in effect of FDI on domestic investments in Bangladesh. By elucidating the mechanisms through which FDI influences domestic investment, the findings offer valuable insights for policymakers and stakeholders seeking to foster sustainable economic development in Bangladesh.

## Limitations & directions for future research

Although this study offers valuable insights, it is important to acknowledge its limitations. The utilization of data only up until 2022 may limit the contemporary relevance of findings, highlighting the necessity for future research with updated data to capture recent trends. Furthermore, the inclusion of supplementary variables and the expansion of the study duration could yield a more thorough comprehension of the interplay between foreign direct investment (FDI) and domestic investment. Future studies should aim to address these limitations to offer more robust insights into the complex relationship between FDI and domestic investment, informing evidence-based policymaking and fostering sustainable economic development.

Notes:

^**1**^https://tcdata360.worldbank.org/indicators/inv.all.pct?country = BRA&indicator = 345&viz = line_chart&years = 1980,2022#

^**2**^The results have been generated in the following ways:

(a) long-run results of the magnitude of crowding effect: (26.73-1)

(b) short-run results of the magnitude of crowding effect:

(7.85 + 5.89+5.43 + 5.39+3.57)**/**[1- (0.23 + 0.21+0.05 + 0.09-0.04)]

For more details, please see appendix 1and appendix 2(page 21–22)

## Funding

No funding was received for this work.

## Additional information

No additional information is available for this paper.

## Data availability statement

Data will be made available on request.

## CRediT authorship contribution statement

**Guo Ai-Jun:** Writing – review & editing, Software, Resources, Methodology, Conceptualization. **Sayed Farrukh Ahmed:** Writing – review & editing, Resources, Methodology, Investigation, Formal analysis. **A.K.M. Mohsin:** Writing – original draft, Visualization, Software, Resources, Investigation, Formal analysis, Conceptualization. **Arifur Rahman:** Writing – review & editing, Resources, Methodology, Formal analysis. **Shamsul Nahar Abdullah:** Writing – review & editing, Resources, Methodology, Formal analysis. **Choo Wou Onn:** Writing – review & editing, Visualization, Methodology, Investigation, Formal analysis. **Mohammad Saiyedul Islam:** Writing – review & editing, Resources, Funding acquisition, Data curation.

## Declaration of competing interest

The authors declare that they have no known competing financial interests or personal relationships that could have appeared to influence the work reported in this paper.

## Figures and Tables

**Table:** 

Vector Error Correction Estimates			
Cointegrating Eq:	CointEq1		
LRGCF(-1)	1.000000		
LRFDI(-1)	26.73083		
	(6.44272)		
	[ 4.07252]		
LRGDP(-1)	−0.861881		
	(0.07857)		
	[-10.9692]		
C	5.160713		
Error Correction:	D(LRGCF)	D(LRFDI)	D(LRGDP)
CointEq1	−0.243141	0.001482	−0.104700
	(0.07143)	(0.00906)	(0.03004)
	[-3.40391]	[ 0.16360]	[-3.48570]

**Table:** 

Dependent Variable: D(LRGCF)				
	Coefficient	Std. Error	t-Statistic	Prob.
C(1)	−0.243141	0.071430	−3.403914	0.0022
C(2)	0.234721	0.136211	1.480946	0.1511
C(3)	0.214854	0.111230	2.210327	0.0365
C(4)	0.054334	0.101040	0.471440	0.6414
C(5)	0.098772	0.046821	1.787041	0.0861
C(6)	−0.040436	0.045710	−0.225229	0.8236
C(7)	7.856231	2.234926	2.531226	0.0071
C(8)	5.892161	2.329278	2.504364	0.0192
C(9)	5.436578	2.313688	2.415632	0.0233
C(10)	5.395432	1.644019	2.739181	0.0112
C(11)	3.578503	1.945189	1.558502	0.1317
C(12)	−1.100395	0.364757	−3.016786	0.0058
C(13)	−0.454206	0.372369	−1.219775	0.2339
C(14)	0.739303	0.291143	2.539313	0.0177
C(15)	0.091622	0.295663	0.309888	0.7592
C(16)	−0.696305	0.253149	−2.750577	0.0109
C(17)	0.074169	0.024038	3.085548	0.0049
